# Myocardial deformation links combined liraglutide–empagliflozin therapy with improved cardiovascular and economic outcomes in type 2 diabetes: a 6-year study

**DOI:** 10.1093/ehjimp/qyag080

**Published:** 2026-04-28

**Authors:** John Thymis, Grigoris Oikonomidis, Georgios Georgiopoulos, George Pavlidis, Dimitris Kourassis, Eleni Katsanaki, Dimitris Vlachomitros, Gavriella Kostelli, Vaia Lambadiari, Ignatios Ikonomidis

**Affiliations:** 2nd Cardiology Department, National & Kapodistrian University of Athens, Attikon University Hospital, Rimini 1, Chaidari, 124 62 Athens, Greece; 2nd Cardiology Department, National & Kapodistrian University of Athens, Attikon University Hospital, Rimini 1, Chaidari, 124 62 Athens, Greece; 2nd Cardiology Department, National & Kapodistrian University of Athens, Attikon University Hospital, Rimini 1, Chaidari, 124 62 Athens, Greece; 2nd Cardiology Department, National & Kapodistrian University of Athens, Attikon University Hospital, Rimini 1, Chaidari, 124 62 Athens, Greece; 2nd Cardiology Department, National & Kapodistrian University of Athens, Attikon University Hospital, Rimini 1, Chaidari, 124 62 Athens, Greece; 2nd Cardiology Department, National & Kapodistrian University of Athens, Attikon University Hospital, Rimini 1, Chaidari, 124 62 Athens, Greece; 2nd Cardiology Department, National & Kapodistrian University of Athens, Attikon University Hospital, Rimini 1, Chaidari, 124 62 Athens, Greece; 2nd Cardiology Department, National & Kapodistrian University of Athens, Attikon University Hospital, Rimini 1, Chaidari, 124 62 Athens, Greece; 2nd Department of Internal Medicine, Research Unit and Diabetes Center, National & Kapodistrian University of Athens, Attikon University Hospital, Rimini 1, Chaidari, 124 62 Athens, Greece; 2nd Cardiology Department, National & Kapodistrian University of Athens, Attikon University Hospital, Rimini 1, Chaidari, 124 62 Athens, Greece

**Keywords:** GLP1-RA, SGLT2i, type 2 diabetes, myocardial deformation, major adverse cardiovascular events, healthcare economics

## Abstract

**Aims:**

We investigated whether the early favourable effects of combined GLP-1 receptor agonist (GLP-1RA) and SGLT2 inhibitor (SGLT2i) therapy in left ventricular deformation is associated with long-term cardiovascular outcomes in patients with type 2 diabetes mellitus (T2DM). We also addressed the healthcare costs.

**Methods and results:**

We enrolled 336 consecutive participants with T2DM, aged 60 ± 10 years old, 252/336 (75%) were males and were categorized into four groups: insulin, liraglutide, empagliflozin, and liraglutide+ empagliflozin. We measured at baseline and at 6 months left ventricular global longitudinal strain (LVGLS) via echocardiography. Patients were followed for 6 years and we recorded the incidence of composite endpoint of non-fatal cardiovascular events (myocardial infarction, heart failure hospitalization, ischaemic stroke, and coronary revascularization). Multivariable Cox regression models were built to assess associations between treatment groups, LVGLS changes, and outcomes. A cost analysis was also conducted. At 6 months LVGLS increased significantly (*P* = 0.020), with the greatest improvement observed in the combination group compared with insulin, GLP-1RA, and SGLT2i groups (*P* < 0.05). At 6-year follow-up, 91 events were recorded. Combination therapy was associated with the lowest risk of events (insulin vs. combination: HR = 4.36, 95% CI: 2.04–9.33, *P* < 0.001; GLP1-RA vs. combination: HR = 2.45, 95% CI: 1.06–5.64, *P* = 0.035; SGLT2i vs. combination: HR = 3.07, 95% CI: 1.38–6.81, *P* = 0.006). In combination group, improvement of LVGLS independently predicted reduced event risk (HR = 0.89, 95% CI: 0.79–0.98, *P* = 0.040). Combination group demonstrated the lowest mean cost per patient per month (insulin: 691€, 95% CI: 472–911; GLP1-RA: 224€, 95% CI: 44–404; SGLT2i: 528€, 95% CI: 305–751; Combination: 175€, 95% CI: 30–321).

**Conclusion:**

Combined GLP-1RA and SGLT2i therapy is associated with early improvement in myocardial deformation, which translates into reduced long-term cardiovascular risk and favourable economic outcomes.

ClinicalTrials.gov Identifier: NCT03878706

## Graphical Abstract

**Graphical Abstract:**
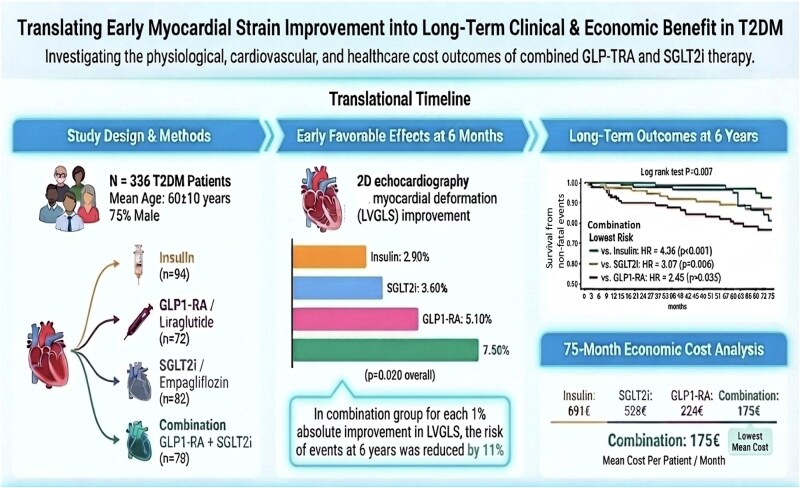

## Introduction

Sodium-glucose cotransporter-2 inhibitors (SGLT2i) and glucagon-like peptide-1 receptor agonists (GLP-1 RAs) are recommended for the management of type 2 diabetes mellitus (T2DM) in patients with elevated cardiovascular risk.^1^ The cardioprotective effects of GLP-1 receptor agonists are primarily mediated through anti-inflammatory and anti-atherogenic mechanisms, while SGLT-2 inhibitors exert several actions, including improvement of vascular haemodynamics and providing beneficial metabolic effects.^1^ We have previously proposed that their combined administration confers synergistically in restoration of myocardial deformation through complementary pleiotropic mechanisms, potentially enhancing therapeutic efficacy.^1^ Markers of myocardial deformation detect early subclinical injury of subendocardial damage and demonstrate significant prognostic value for the future development of major adverse cardiovascular events.^2^Nonetheless, whether the favourable effects of the combination on left ventricular deformation is associated with reduced incidence of future adverse cardiovascular events in high-risk diabetics has not been fully addressed yet. Additionally, we investigated the healthcare economic outcomes of the dual treatment compared with the separate administration of each agent and the treatment with insulin.

## Methods

We totally recruited 336 consecutive individuals with established T2DM who attended our outpatient Cardiometabolic Clinic. Participants underwent a comprehensive clinical evaluation and documented a detailed medical history assessment at baseline. Participants of the current study were included in our previous studies^1^ and were categorized into four treatment groups: insulin group (*n* = 94), GLP1-RA liraglutide group (*n* = 72), SGLT2i empagliflozin group (*n* = 82), and the combination GLP1-RA + SGLT2i group (*n* = 78), using either randomization^3^ or propensity score matching, as previously described.^1^ We conducted in all participants a complete echocardiographic study at recruitment and at 6 months. Speckle-tracking echocardiography was performed using dedicated software (EchoPac206, GE Healthcare, Horten, Norway) to assess myocardial deformation. Left ventricular global longitudinal strain (LVGLS) was calculated from two-dimensional echocardiographic images acquired at a frame rate of 70–80 frames/s from the apical four-, two-, and three-chamber views, according to the standardized 17-segment left ventricular model and manual correction of tracing was used if appropriate.^1^

All participants were followed-up for 6 years and we strictly recorded the occurrence of the composite outcome of adverse non-fatal cardiovascular events consisting of: myocardial infarction, heart failure hospitalization, ischaemic stroke, and coronary revascularization for chronic coronary syndromes.

We conducted repeated measures ANOVA for (i) measurements of the examined GLS at baseline and at 6 months of treatment, which was considered as a within-subject factor (the respective F and *P* values are reported) and (ii) for the effects of different treatments, as a between-subject factor (the respective F and *P* values for the interaction of treatment × time are reported). We also applied one-way ANOVA with *post hoc* pairwise comparisons using Bonferroni corrections. Time-to-event analyses were performed by employing multivariable Cox proportional hazards regression models to assess the association between the four treatment groups and the incidence of the composite cardiovascular outcome. Between-group comparisons were conducted by including treatment group as a categorical variable in the models, with the combination therapy group used as the reference category. The models were adjusted for baseline age, sex, hypertension, hyperlipidaemia, smoking, coronary artery disease (CAD), body mass index (BMI), and glycosylated haemoglobin (HbA1c). Hazard ratios (HRs) with corresponding 95% confidence intervals (CIs) are provided. Also, multivariable Cox models adjusted for age, sex, hypertension, hyperlipidaemia, smoking CAD, BMI, and HbA1c were built to assess the association of the absolute GLS change (percentage points) with the incidence of events. Event-free survival across the four treatment groups was estimated using Kaplan–Meier survival analysis, and survival curves were constructed for each group, with between-group differences assessed using the log-rank test. Risk tables report the respective number of patients at risk at predefined time intervals and to support the graphical representation of the survival curves. A cost analysis was performed over a fixed 75-month horizon using one observation per patient. Drug costs were assigned according to treatment group and accumulated over follow-up (300 €/month for groups of insulin, GLP1-RA, SGLT2i, and 600 €/month for the combination group). Event-related costs were applied for myocardial infarction (2976.36 €), heart failure hospitalization (1011.50 €), revascularization (2.001,12€), and stroke (2317.68 €) when these events occurred within the 75-month period, considering only the first occurrence of each event. For each participant cumulative, event-related costs were calculated as the sum of the corresponding unit costs (or zero if no event occurred). Group-specific mean cumulative costs per patient were compared using ordinary least squares (OLS) regression, with treatment group as the main exposure and adjustment for age and sex, applying robust (Huber–White) standard errors. Adjusted mean costs and 95% confidence intervals for each group were estimated using marginal standardization. To verify robustness, we additionally examined event counts by group and confirmed that the width of the confidence intervals closely tracked the number of cost-incurring events. Statistical analyses were conducted using SPSS version 30 (IBM SPSS Statistics, IBM Corp., Chicago, IL, USA) and Stata version 18.0 (StataCorp LLC, College Station, TX, USA). The data supporting the study findings will be available from the corresponding author upon reasonable request.

## Results

The median follow-up period was 69 (60–75) months, 252/336 (75%) were males and the mean age of our population was 60 ± 10 years. The 134/336 (40%) had CAD, 225/336 (67%) hypertension, 171/336 (50.8%) smoking, and 336/336(100%) hyperlipidaemia. No significant differences in the prevalence of risk factors was observed between the four treatment groups (*P* > 0.05) (*Table 1*). Baseline LVGLS was −16.7 ± 3.1% and no difference was observed between the treatment groups (*P* > 0.05). At 6-month follow-up, significant improvement of GLS was noted in our sample (from −16.7 ± 3.1% to −17.5 ± 3.9%, *F* = 5.03, *P* = 0.020). A significant interaction was observed between change of GLS and treatment groups (*F* = 4.63, *P* = 0.028). Participants under the combination demonstrated the greatest percent improvement of LVGLS compared with the other groups (combination group: 7.50% [95% CI: 2.90–12.10] vs. insulin group: 2.90% [95% CI: 0.70–5.10], *P* = 0.010; vs. GLP1-RA group: 5.10% [95% CI: 1.20–6.90], *P* = 0.039; vs. SGLT2i group: 3.60% [95% CI: 0.60–6.60], *P* = 0.024 respectively). Baseline LV GLS did not affect LV GLS change at follow-up (*P* > 0.05).

**Table 1:** Baseline clinical characteristics of the study population

	All patients (*n* = 336)	Insulin (*n* = 94)	Liraglutide (*n* = 72)	Empagliflozin (*n* = 92)	Liraglutide + Empagliflozin (*n* = 78)	*P*
Age, years	60 ± 10	61 ± 10	59 ± 9	58 ± 11	59 ± 10	0.676
Sex (male/female), *n* (%)	252/84 (75/25)	70/24 (74.5/25.5)	54/18 (75/25)	64/28 (69/31)	64/14 (82/18)	0.316
Risk factors, *n* (%)						
Coronary artery disease	134 (40)	38 (40.4)	29 (40.3)	33 (35.8)	34 (43.6)	0.781
Current smoking	171 (50.8)	48 (51.1)	36 (50)	42 (45.6)	45 (57.7)	0.479
Hyperlipidaemia	336 (100)	94 (100)	72 (100)	92 (100)	78 (100)	1.000
Hypertension	225 (67)	64 (68.1)	48 (66.7)	55 (59.8)	58 (74.3)	0.248

Data are presented as mean ± SD, number (percentage), or median (first quartile–third quartile). Scale variables were compared using one-way ANOVA. Binary variables were compared with the χ^2^ test.

**Figure 1:**
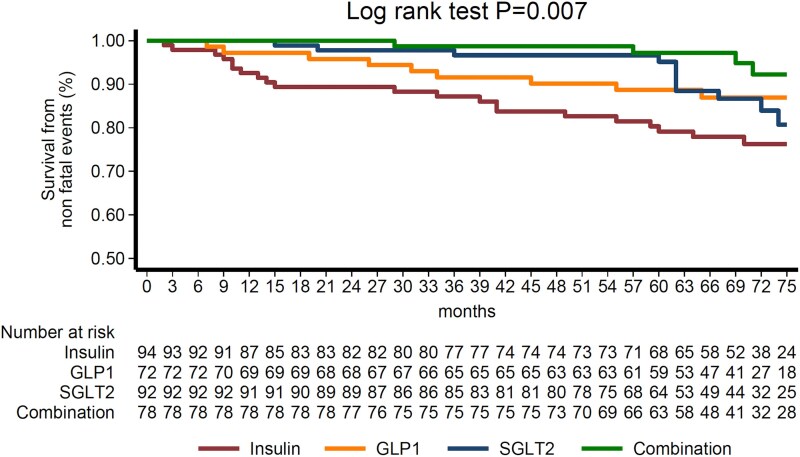
Kaplan–Meier survival analysis. Event-free survival across the four treatment groups was estimated using Kaplan–Meier survival analysis, and survival curves were constructed for each group, with between-group differences assessed using the log-rank test. Risk tables report the respective number of patients at risk at predefined time intervals and to support the graphical representation of the survival curves.

At 6-year follow-up, *n* = 91 non-fatal events were documented, of which (*n* = 22) myocardial infarctions, (*n* = 23) heart failure hospitalizations, (*n* = 7) strokes, and (*n* = 39) coronary revascularizations. The combination group displayed the lowest risk for developing the composite outcome of non-fatal cardiovascular events (insulin vs. combination group: HR = 4.36, 95% CI: 2.04–9.33, *P* < 0.001; GLP1-RA vs. combination group: HR = 2.45, 95% CI: 1.06–5.64, *P* = 0.035; SGLT2i vs. combination group: HR = 3.07, 95% CI: 1.38–6.81, *P* = 0.006) (*Table 2*) (*Figure 1*). In the combination arm, the percent change of LVGLS at 6 months was independently associated with the incidence of the composite outcome (HR: 0.89, 95% CI: 0.79–0.98, *P* = 0.040).

**Table 2:** Multivariable Cox proportional hazard models of antidiabetic treatments for the composite outcome

Group comparison (Reference: combination)	Hazard ratio (HR)	95% confidence intervals	*P*-value
Insulin vs. Combination	4.36	2.04–9.33	<0.001
Liraglutide (GLP-1RA) vs. combination	2.45	1.06–5.64	0.035
Empagliflozin (SGLT2i) vs. combination	3.07	1.38–6.81	0.006

Incidence of non-fatal cardiovascular events at 6-year follow-up					
Outcome	All patients (*n* = 336)	Insulin (*n* = 94)	Liraglutide (*n* = 72)	Empagliflozin (*n* = 92)	Liraglutide+ Empagliflozin (*n* = 78)
Non-fatal CV events (composite)	91 (27.1%)	40 (42.5%)	18 (25%)	25 (27.2%)	8 (10.3%)
Myocardial infarction	22 (6.3%)	12 (12.7%)	3 (4.2%)	5 (5.4%)	2 (2.6%)
Heart Failure Hospitalization	23 (6.8%)	10 (10.6%)	6 (8.3%)	5 (5.4%)	2 (2.6%)
Stroke	7 (2.1%)	3 (3.2%)	1 (1.4%)	2 (2.2%)	1 (1.3%)
Coronary Revascularization	39 (13.1%)	15 (20.8%)	8 (14.3%)	13 (14.1%)	3 (3.8%)

Multivariable Cox regression models adjusted for age, sex, hypertension, hyperlipidemia, smoking, Coronary Artery Disease, Body Mass Index and glycosylated Haemoglobin were built to identify the association between the antidiabetic treatment groups and the incidence of the composite outcome during 6-year follow-up. The composite outcome of adverse non-fatal cardiovascular events consists of: myocardial infarction, heart failure hospitalization, ischemic stroke and coronary revascularization for chronic coronary syndromes. Combination group was used as a reference category. The Hazard ratio, along with the respective 95% Confidence Intervals is presented. Also, the incidence of non-fatal events at 6-year follow-up are presented as absolute and relative frequencies.

The cost estimation of hospitalization for non-fatal CV events revealed that participants in the combination group displayed the lowest total cost per patient per month through 75 months (insulin: 691€, 95% CI: 472–911; GLP1-RA: 224€, 95% CI: 44–404; SGLT2i: 528€, 95% CI: 305–751; combination: 175 €, 95% CI: 30–321).

Only 15/336 (4.46%) participants had changed their medications through follow-up. After performing a sensitivity analysis, no change of our results was observed.

## Discussion

In the current study, short-term amelioration of myocardial deformation in type 2 diabetics provided from co-administration of liraglutide and empagliflozin was independently associated with reduced major cardiovascular events incidence long-term and in turn in better economic outcomes. Our study findings are aligned with other reports from cohorts regarding the beneficial effects of the combined therapy in reducing incidence MACEs.^4^ Interestingly, our study had a relatively larger follow-up period and also our current results imply that improvement in myocardial performance may be a fundamental pathophysiological link between the cardioprotective combined treatment and long-term clinical benefit. A study evaluating SGLT2i vs. sitagliptin demonstrated neutral efficacy in LV GLS change at 6 months but SGLT2i conferred a substantial improvement in participants with baseline LVGLS > -16.4.^5^ In our study, we demonstrated significant improvement of GLS in high-risk diabetics treated with SGLT2 inhibitors who had a mean GLS value of approximately 16.7%. However, the combination group GLP1 + SGLT2i demonstrated the most profound improvement, and this change was associated with improved outcomes at follow up. LVGLS constitutes a sensitive marker of subclinical myocardial dysfunction, capable of detecting early impairment in myocardial contractility before a decline in left ventricular ejection fraction becomes overt.^2^ This is especially relevant among diabetics, in whom the subendocardial longitudinal fibres—highly susceptible to coronary microcirculatory dysfunction, ischaemia, metabolic stress, and increased arterial stiffness—are affected early in the disease process.^2^ Studies support that increased arterial stiffening in patients with CAD or stroke may be related to impaired GLS through early arrival of reflected waves leading to increased central aortic systolic pressure (and thus increased oxygen demand) and reduced central diastolic pressure (and thus reduced myocardial perfusion).^6^ In this context, the greater amelioration of LVGLS observed with combined therapy may reflect an early reversal of subclinical myocardial injury. Importantly, LVGLS has been shown to provide incremental prognostic value long-term beyond traditional cardiovascular risk scores, such as Framingham and SCORE.^2^ Consequently, the assessment of left ventricular deformation may serve prognostically for risk stratification and monitoring the efficacy of combined GLP-1RA and SGLT2i treatment. Our study also confers novel evidence regarding the robust economic outcomes derived by the prolonged administration of the dual treatment. The following limitations apply to our study as well. Firstly, we conducted a single centre study with relatively small sample size. Also, due to high incidence of revascularizations in our sample the cost analysis may be affected by the procedures. Additionally, participants in our cohort were treated exclusively with liraglutide among GLP-1 receptor agonists and empagliflozin among SGLT2 inhibitors, which limits the interpretation of potential class-specific differences. Larger prospective cohorts are warranted to validate our findings.

## Conclusion

Administration of combined GLP-1RA and SGLT2i therapy leads to early improvement in myocardial deformation and is associated with a mitigated long-term risk of major adverse cardiovascular events (MACE). Additionally, this dual regimen appears to confer favourable economic outcomes.

## Data Availability

The data underlying this article will be shared on reasonable request to the corresponding author.

## Lead author biography

Dr Ignatios Ikonomidis, MD, PhD, is Professor of Cardiology, Director of Echocardiography, Laboratory of Preventive Cardiology and Clinic of Cardiometabolic Diseases, 2nd Cardiology Department, National and Kapodistrian University of Athens (NKUA), Attikon Hospital, Athens, Greece. In 1997, he completed his training in Cardiology and Cardiac Imaging, in Hammersmith Hospital, Imperial College, London, UK. Since then, he worked as consultant cardiologist in University Hospital of Patras, Department of Clinical Therapeutics and 2nd Cardiology Department, Medical School, Athens. His research fields include cardio-vascular imaging, microcirculation, inflammation, diabetes, smoking control, and ventricular–arterial interaction. He has authored 354 publications in indexed journals and several book chapters.
